# Fishing intensification as response to Late Holocene socio-ecological instability in southeastern South America

**DOI:** 10.1038/s41598-021-02888-7

**Published:** 2021-12-06

**Authors:** Alice Toso, Ellen Hallingstad, Krista McGrath, Thiago Fossile, Christine Conlan, Jessica Ferreira, Dione da Rocha Bandeira, Paulo César Fonseca Giannini, Simon-Pierre Gilson, Lucas de Melo Reis Bueno, Murilo Quintans Ribeiro Bastos, Fernanda Mara Borba, Adriana M. P. do Santos, André Carlo Colonese

**Affiliations:** 1 Department of Prehistory and Institute of Environmental Science and Technology (ICTA), Universitat Autònoma de Barcelona, 08193 Bellaterra, Spain; 2 BioArCh, Department of Archaeology, University of York, York, YO10 5DD UK; 3 Department of Archaeology, Simon Fraser University, Education Building 9635, 8888 University Dr., Burnaby, BC V5A 1S6 Canada; 4 Departamento de Ciências Biológicas - Meio Ambiente e Biodiversidade, Rua Paulo Malschitzki 10, Zona Industrial Norte, Universidade da Região de Joinville, Joinville, Santa Catarina 89219-710 Brazil; 5 Museu Arqueológico de Sambaqui de Joinville, Programa em Patrimônio Cultural e Sociedade, Universidade da Região de Joinville, Joinville, Brazil; 6 Instituto de Geociências, Universidade de São Paulo, Rua Do Lago, 562, São Paulo, 05508-080 Brazil; 7 Instituto de Ciências Humanas e da Informação, Universidade Federal do Rio Grande, Rio Grande, Brazil; 8 Departamento de História, Laboratório de Estudos Interdisciplinares em Arqueologia (LEIA), Universidade Federal de Santa Catarina, Florianópolis, Brazil; 9 Departamento de Antropologia, Museu Nacional, Universidade Federal do Rio de Janeiro, Rio de Janeiro, Brazil

**Keywords:** Biological techniques, Molecular biology, Ecology, Biomarkers

## Abstract

The emergence of plant-based economies have dominated evolutionary models of Middle and Late Holocene pre-Columbian societies in South America. Comparatively, the use of aquatic resources and the circumstances for intensifying their exploitation have received little attention. Here we reviewed the stable carbon and nitrogen isotope composition of 390 human individuals from Middle and Late Holocene coastal sambaquis, a long-lasting shell mound culture that flourished for nearly 7000 years along the Atlantic Forest coast of Brazil. Using a newly generated faunal isotopic baseline and Bayesian Isotope Mixing Models we quantified the relative contribution of marine resources to the diet of some of these groups. Through the analysis of more than 400 radiocarbon dates we show that fishing sustained large and resilient populations during most of the Late Holocene. A sharp decline was observed in the frequency of sambaqui sites and radiocarbon dates from ca. 2200 years ago, possibly reflecting the dissolution of several nucleated groups into smaller social units, coinciding with substantial changes in coastal environments. The spread of ceramics from ca. 1200 years ago is marked by innovation and intensification of fishing practices, in a context of increasing social and ecological instability in the Late Holocene.

## Introduction

The origin and development of plant-based economies have dominated evolutionary models of economic intensification in pre-Columbian South America, and influenced attempts to explain changes in the social organization, population growth and emergence of social complexity across most of the continent during the Late Holocene^1–4^. Agriculture, in particular, is often regarded as the prime mover of major cultural and demographic changes in the pre-Columbian era^5–7^. However, it is also known that mixed economies founded on plant gathering-cultivation (wild and domesticated), hunting and fishing, prevailed in several cultural hotspots, igniting and sustaining many of the socio-economic traits that are typically associated with the development of agriculture^8^, such as population growth, social complexity, and decreasing residential mobility. Some of these systems involved substantial dependence on aquatic resources in areas considered peripheral to early centres of plant cultivation^9–14^.

The southern Atlantic Forest coast of Brazil preserves archaeological evidence of mixed economies that endured over extensive geographic areas during the Middle and Late Holocene. Human groups have exploited the region’s coastal resource systems since at least 7000 years ago^15^, as attested by numerous cultural shell mounds, locally known as sambaquis ("mountain of shells'' in the Tupi language)^11, 12, 16–19^. Sambaquis were concentrated around resource-rich coastal environments (bays, estuaries and lagoons) where relatively abundant and predictable aquatic resources offered conditions for the establishment of dense and stable populations, potentially with similar or shared cultural identities in some areas^11, 12, 15, 20, 21^. Studies have demonstrated that several large shell mounds were occupied for extensive periods of time^22, 23^ as collective or community-based cemeteries with enduring political and ideological values^11, 12, 15, 24^. Parallel to these large sites, smaller and shallower sites similarly offer faunal remains depicting a high degree of dependence on aquatic resources^16–19, 25–29^. Stable carbon (δ^13^C) and nitrogen (δ^15^N) isotope analyses of bone collagen of hundreds of human individuals indicate that aquatic organisms were the primary source of dietary proteins^23, 30–40^. While plant resources were unarguably important and possibly secured through a combination of gathering, management, and cultivation of wild and domesticated crops^31, 41–43^, the exploitation of aquatic resources appears to have been central to the subsistence economy and beyond.

In southern Brazil, an apparent rupture with this socio-ecological system occurred from ca. 2000 years ago, as indicated by a decline in radiocarbon dates and noticeable changes in site formation processes, such as the appearance of distinctive, dark humic deposits with abundant fish remains at the top of large pre-existing sites or in shallow sites known as “late sambaquis”^25, 28, 44^. Previous works have attributed the demise of large shell mounds to a combination of factors, involving changes in coastal environments^45^ as well as the expansion of the highland horticulturists, known as the Taquara-Itararé or proto-Jê tradition in southern Brazil (hereafter referred to as Taquara-Itararé), who would have introduced ceramics and new crops to the coast^11, 12, 15, 46, 47^. Stable isotope analysis of bone collagen has been crucial in questioning the degree of dietary and economic change during the introduction of ceramics, showing a continuation of coastal exploitation in some areas^30, 32, 33^. However, the generalized lack of regional isotopic baselines has prevented robust dietary interpretations and exacerbated uncertainties about the stability and diversity of subsistence systems at this time. Moreover, the discontinuous chronology of sambaqui sites and the geographic variability in radiocarbon dates have hampered attempts to determine the nature, scale and timing of this transformative process.

**Figure 1:**
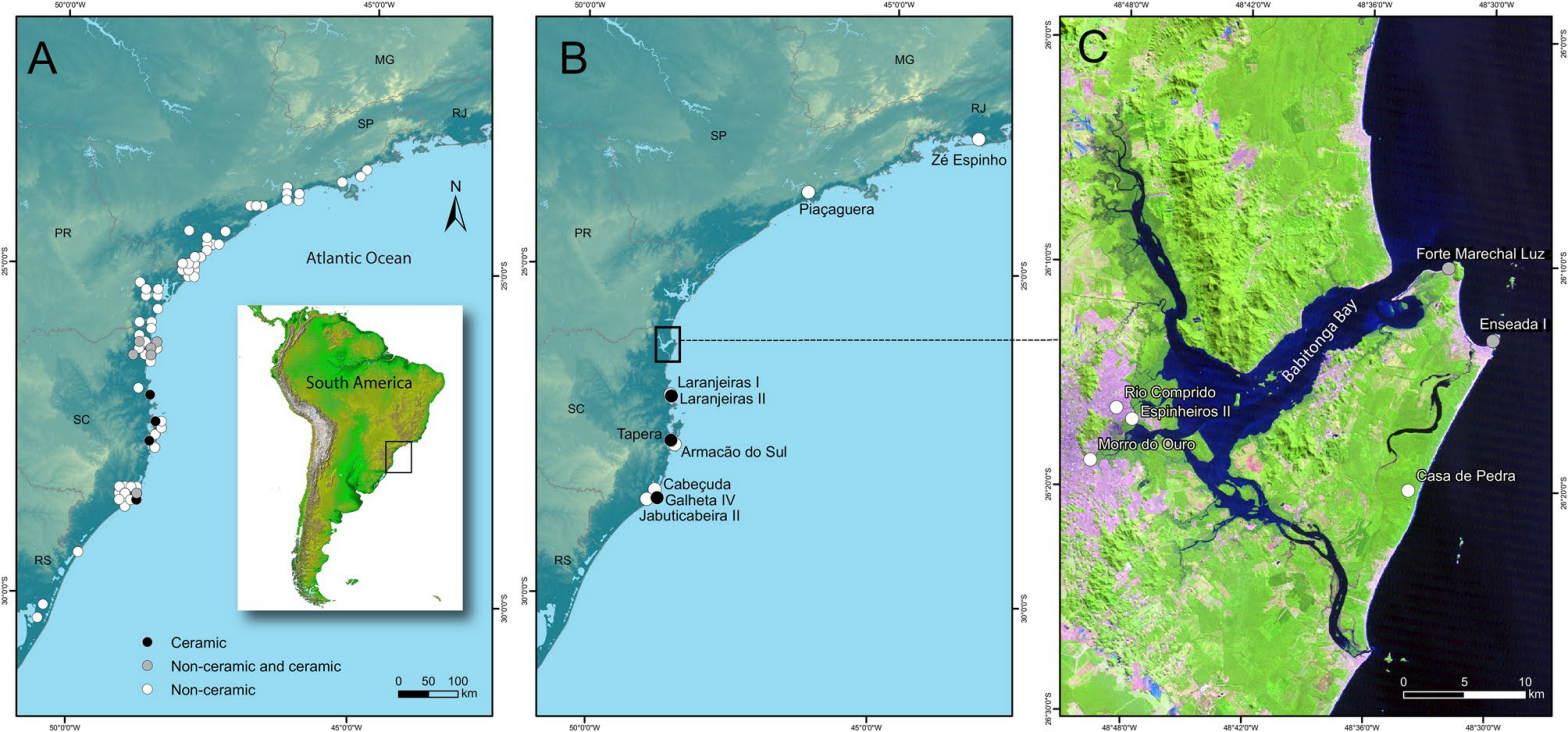
(**A**) Location of non-ceramic and ceramic sites with radiocarbon dates analysed in this study, (**B**) Location of non-ceramic and ceramic sites in southeast and south of Brazil used for stable isotope analysis, and (**C**) site distribution in Babitonga Bay (Santa Catarina). Maps generated using ArcGIS 10.7 (https://desktop.arcgis.com/en/), Inkscape (https://inkscape.org/pt-br/), Adobe Illustrator CS6 (https://www.adobe.com/es/products/illustrator.html), and GNU Image Manipulation Program (https://www.gimp.org/), on data publicly available from DIVA-GIS (http://www.diva-gis.org/gdata), Natural Earth (https://www.naturalearthdata.com/), Brazilian Institute of Geography and Statistics (IBGE), and National Institute for Space Research (INPE) and NASA/JPL/NIMA (South American).

In this work we reassess the importance of aquatic resources to the subsistence economies of pre-Columbian groups along the southeastern and southern coasts of Brazil during the Late Holocene, prior to and after the introduction of Taquara-Itararé ceramics to the coast, during a period of increasing climate, environmental and social instability. We focused our analysis on sites from Babitonga Bay (northern Santa Catarina state in southeastern Brazil, Fig. 1), a region with the largest concentration of sambaquis on the Brazilian coast^48, 49^. We reassessed the dietary significance of δ^13^C and δ^15^N values of pre-Columbian individuals with the support of a newly generated local faunal isotope baseline and Bayesian Isotope Mixing Models (BSIMM). For comparative purposes we reviewed the existing stable isotope data for human and faunal samples from the southeastern and southern Brazilian coasts, and discussed their dietary interpretation within a new chronological model developed with over four hundred aggregated radiocarbon dates for coastal non-ceramic and early ceramic sites.

## Results

### Chronological models

**Figure 2:**
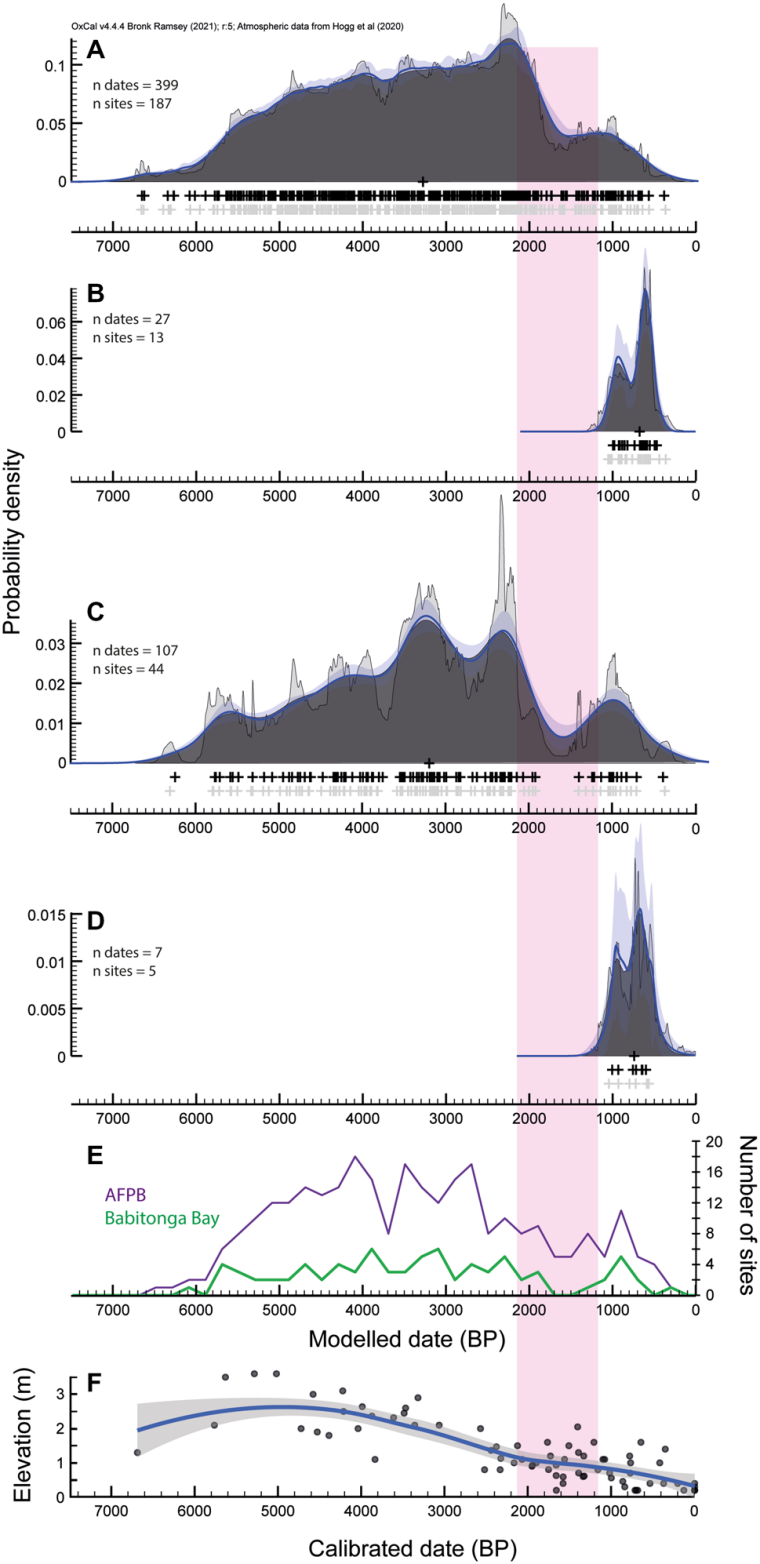
KDE model for (**A**) non-ceramic sites in the AFPB; (**B**) ceramic sites/stratigraphic contexts in Santa Catarina state; (**C**) non-ceramic sites in Babitonga Bay; (**D**) ceramic sites/stratigraphic contexts in Babitonga Bay. The dark gray distribution corresponds with the KDE estimated distribution. The medians of the likelihood distributions from the simulated ^14^C measurements and the medians of the marginal posterior distributions for the events from the KDE model analysis are represented by the light gray and black crosses respectively. The mean ± 1σ for snapshots of the KDE distribution generated during the Markov chain Monte Carlo (MCMC) process are represented by the overlying blue line and lighter blue band^50^. (**E**) Frequency of non-ceramic sambaquis sites for the AFPB and Babitonga Bay using 200-year bin; (**F**) aggregated relative sea level fluctuation for southern Brazil^45, 53^. The red bar represents the time interval between the decline in radiocarbon dates of non-ceramic sites and the earliest evidence for Taquara-Itararé ceramics in coastal sites.

Aggregated calibrated radiocarbon dates and their associated chronological uncertainty provide insights into the frequency and intensity of past human activities^50–52^. In the case of non-ceramic sambaquis, the Kernel Density Estimation model (KDE) outputs from the Atlantic Forest and the northern Pampa Biome (hereafter denoted as AFPB), and Babitonga Bay show a series of oscillations from ca. 6600 to 400 cal BP, with most of the sites formed between ca. 5500 and 2200 cal BP (Fig. 2A,C,E). The highest density of dated sites and ^14^C dates coincide with a period of high relative sea level and its steady fall along the southern Brazilian coast^45, 53^ (Fig. 2F). The frequency of dated sites shows a gradual decreasing trend from ca. 2500, followed by a sharp decline in both dated sites and radiocarbon distribution at ca. 2200 cal BP. This rupture in the system coincides with changes in coastal environments in southern Brazil associated with falling sea level^45, 53^, increasing precipitation and freshwater input^54, 55^, and an abrupt increase in cold frontal depressions^56^. This allows for broadly discussing the archaeological record in the context of an early (ca. > 2000 cal BP) and late (ca. < 2000 cal BP) non-ceramic period. This sudden decline predates by ca. 1000 years the earliest modelled dates for the introduction of Taquara-Itararé ceramics on the coast (1150–900 cal BP at Galheta IV and 1200–850 at Enseada I, both at 95.4% confidence interval) (Fig. 2B–D). The KDE model outputs for non-ceramic sambaqui sites had good general agreements for both AFPB and Babitonga Bay, with A_model_ of 69.6 and 119.7 respectively, which suggest both models were robust (Supplementary Information 1).

Similar to non-ceramic sites, the model outputs for ceramic sites attributed to the Taquara-Itararé tradition had good agreements, with A_model_ of 114.8 and 110.4 for Santa Catarina state and Babitonga Bay, respectively. The chronological models for the dispersal of Taquara-Itararé ceramics show a bimodal distribution in both regions (Fig. 2B–D). The first and minor peaks are centered around 1000 cal BP, followed by a more pronounced distribution centered around 700 cal BP. Overall, the chronological models suggest that the dispersal of Taquara-Itararé ceramics to the coast was a two-step process, with an incipient and localized introduction event ca. 1200–900 years ago followed by a more pronounced dispersal process ca. 800–600 years ago, coinciding with the demographic boom of the Taquara-Itararé population in the southern Brazilian highlands^57^.

### Review of human and faunal stable isotope values

Our review of the literature identified bone and dentine collagen δ^13^C and δ^15^N values for a total of 390 human samples from non-ceramic and ceramic contexts in southern and southeastern Atlantic Forest coast of Brazil. Of these, 268 (69%) met our selection criteria and were considered in this study. These consisted of 180 samples from non-ceramic contexts (n = 12) and 88 samples from ceramic contexts (n = 5) located in coastal areas (estuaries, bays, lagoons and coastal sand barriers) between latitudes 22.9°S and 28.5°S (Supplementary Information 2). Non-ceramic contexts (Enseada I, Armação do Sul, Cabeҫuda, Espinheiros II, Forte Marechal Luz, Jabuticabeira II, Laranjeiras I, Morro do Ouro, Piaçaguera, Rio Comprido, Zé Espinho, Casa de Pedra) had modelled radiocarbon dates between 5950–5550 (Casa de Pedra^22^) and 1300–500 cal BP (Forte Marechal Luz^58^), while ceramic contexts (Enseada I, Forte Marechal Luz, Galheta IV, Laranjeiras II, Praia da Tapera) had radiocarbon dates ranging between 1200–850 (Enseada I^36, 47^) and 750–500 cal BP (Galheta IV^28^) at 95.4% probability. The δ^13^C and δ^15^N values were mostly produced on bone collagen of individuals of distinct sexes and ages (n = 222), followed by dentine collagen (n = 46), in some cases from individuals whose bone collagen had also been analyzed (e.g. Morro do Ouro). One human sample from the non-ceramic site of Cabeçudas had an atomic C:N ratio of 3.66, however, its δ^13^C and δ^15^N values were consistent with other individuals from the same site and therefore this sample was considered reliable. Similarly, two samples from Enseada I had C:N ratios of 2.53 and 2.75, but their δ^13^C and δ^15^N values were comparable with individuals from contemporary sites in Babitonga Bay (e.g. Espinheiros II), and so were considered in the analysis.

Regarding faunal remains, only a limited number of archaeological specimens (n = 39) from a few non-ceramic (n = 4) and ceramic (n = 2) contexts met the quality criteria and were included in this study^30, 32, 59^. These consisted of terrestrial mammals (n = 24), marine fish (n = 6), seabirds (n = 4), sea mammals (n = 3), freshwater crocodile (n = 1) and river otter (n = 1). These organisms were grouped into three broad categories: terrestrial (terrestrial mammals), marine-brackish (marine fish, seabirds, sea mammals) and freshwater (freshwater crocodile and river otter) (Supplementary Information 2).

**Figure 3:**
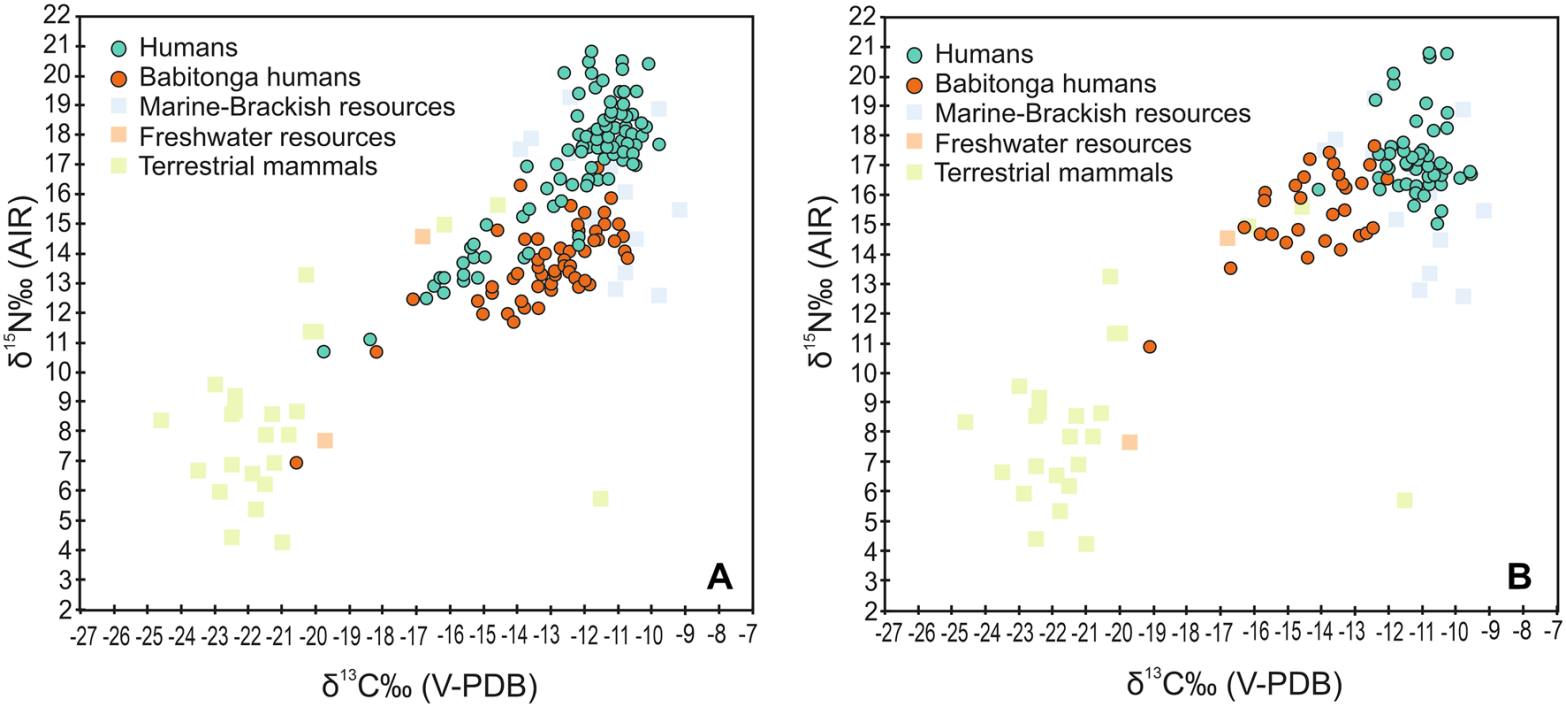
Bulk collagen δ^13^C and δ^15^N values of faunal and human samples from non-ceramic (**A**) and ceramic sites (**B**) in the Atlantic Forest coast of Brazil. “Babitonga humans” refers to individuals from sites located in Babitonga Bay, while “humans” refers to individuals from sites located along the southern Atlantic Forest coast of Brazil.

**Figure 4:**
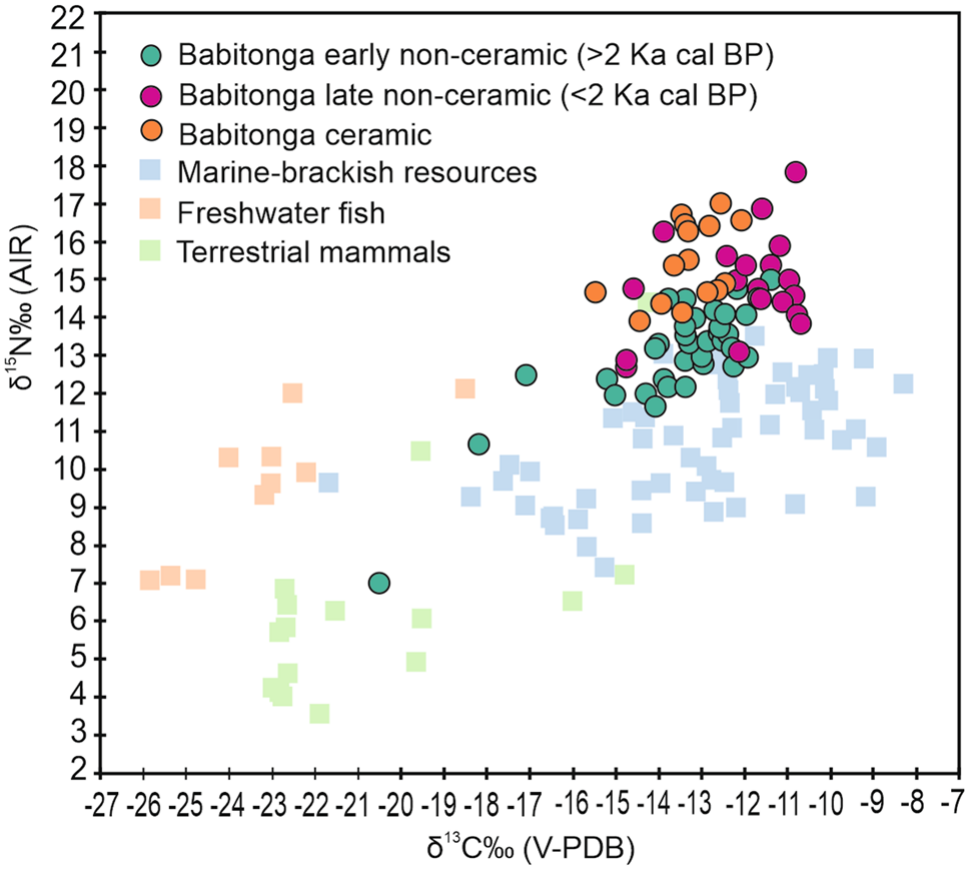
Bulk collagen δ^13^C and δ^15^N values of Middle and Late Holocene human and faunal samples from Babitonga Bay. Human isotopic values are distinguished between early non-ceramic, late non-ceramic, and ceramic populations. For details see supplementary information 2.

The δ^13^C and δ^15^N values of human individuals from non-ceramic contexts ranged from − 20.6 to − 9.8‰ and from + 20.8 to + 7.0‰, respectively. A smaller variation was found among individuals from ceramic contexts, with δ^13^C and δ^15^N values ranging from − 19.2 to − 9.6‰ and from + 20.8 to + 10.9‰, respectively. Comprehensively, the δ^13^C and δ^15^N values of non-ceramic and ceramic groups from the southern and southeastern Atlantic Forest coast (including Babitonga Bay) were statistically indistinguishable (Kruskal–Wallis Test *p* = 0.3223 and 0.5024 for δ^13^C and δ^15^N respectively), and predominantly distributed on the range of values for marine-brackish fauna (Fig. 3A,B). When considering the individuals excavated from sites in Babitonga Bay, the results show that both non-ceramic and ceramic groups had δ^13^C and δ^15^N values comparatively lower than populations from other coastal areas of the southern and southeastern Atlantic Forest. Differences were also found between non-ceramic and ceramic groups from Babitonga Bay, with non-ceramic groups having δ^15^N values significantly lower than their ceramic counterparts (Kruskal–Wallis Test, *p* < 0.0001), although their δ^13^C values were statistically indistinguishable (Kruskal–Wallis, *p* = 0.1607). Moreover, late non-ceramic individuals postdating the decline of sambaquis (< 2000 cal BP; including Enseada I, Forte Marechal Luz, Espinheiros II) have δ^15^N and δ^13^C values significantly higher (Kruskal–Wallis Test, *p* = 0.0003 and *p* = 0.0003) than early non-ceramic individuals (> 2000 cal BP; Morro do Ouro, Rio Comprido, Casa de Pedra), and indistinguishable from ceramic groups for δ^15^N (Kruskal–Wallis Test, *p* = 0.643), although some differences emerged in δ^13^C values (Kruskal–Wallis Test, *p* = 0.003) as reported by previous studies^33^, which may concerns mostly the energy contribution to diet (Fig. 4). In conclusion, the temporal variability in human δ^13^C and δ^15^N values suggests an increased consumption of high trophic level marine organisms in Babitonga Bay from ca. 2000 years ago.

### δ^13^C and δ^15^N values of faunal remains from Babitonga bay

Bulk collagen was successfully extracted from 90 out of 143 faunal samples (63% of total samples) from sites in Babitonga Bay. In 99% of the samples (n = 89) their wt%C and wt%N ranged from 13.7 to 56.1 and from 4.5 to 16, respectively, and exhibited C:N molar ratios from 2.90 to 3.60. Overall, the atomic composition indicated adequate collagen preservation for most samples. One sample had a wt%N of 19.7 and another had C:N molar ratio of 3.70 but their δ^13^C and δ^15^N values were consistent with other samples from the same site (Supplementary Information 2).

Marine-brackish water fish (n = 50; Morro do Ouro, Cubatão I, Bupeva II, Casa de Pedra) had δ^13^C values ranging from − 18.4 to − 8.3‰ (average − 12.8 ± 2.3‰), and δ^15^N values from + 7.4 to + 13.6‰ (average + 11.1 ± 1.5‰). The δ^13^C values reflect assimilated carbon from benthic microalgae and marine phytoplankton^60^, while the δ^15^N values indicate distinct trophic positions, from piscivore-invertivore taxa (e.g. *Trichiurus* sp.) to omnivore species feeding on invertebrates and occasionally fish (e.g. Tetraodontidae, *Micropogonias furnieri*), depending on age and season. The δ^15^N values are comparable with values of juvenile specimens inhabiting mangrove systems, which are lower than their adult counterparts from deeper habitats (ocean, coastal bays) due to ontogenetic differences in feeding behaviour and trophic positions^61, 62^. Marine mammal samples (n = 13; Morro do Ouro, Bupeva II and Casa de Pedra) identified through collagen peptide mass fingerprinting included *Eubalaena australis*, *Megaptera novaeangliae* and oceanic dolphins (Delphinidae). Their δ^13^C and δ^15^N values comprehensively ranged from − 21.7 to − 9.2‰ (average − 14.2 ± 3.7‰) and + 8 to + 13.2‰ (average + 10.3 ± 1.83‰), respectively.

Freshwater fish (n = 10, Itacoara) had δ^13^C values ranging − 25.9 to − 18.5‰ (average − 23.3 ± 2.0‰), and δ^15^N values ranging from + 7.1 to + 12.1‰ (average + 9.5 ± 1.8‰). The δ^13^C values are consistent with the influence of organic matter derived from C_3_ vegetation and phytoplankton in freshwater systems^60^, even though it is important to emphasize that fish δ^13^C and δ^15^N values in freshwater and marine systems are controlled by more complex mechanisms. For example, environmental conditions that affect primary producers at the base of the food web (pH values, availability of CO_2,_ temperature, oxygenation, water movement) impact carbon isotopes^63^, while nitrogen isotopes are also controlled by the isotopic composition of dissolved inorganic/organic nitrogen (DIN/DON) at the base of the food web from which all subsequent isotope fractionations occur (bacterially mediated or trophic enrichment)^64, 65^.

Terrestrial faunal remains (n = 17; Morro do Ouro, Cubatão I, Itacoara) produced δ^13^C values ranging from − 23.0 to − 14.3‰ (average − 20.7 ± 2.9‰) and δ^15^N values ranging from + 3.6 to + 14.4‰ (average + 6.2 ± 2.6‰). It is worth noting the unexpectedly high δ^13^C and δ^15^N values of two coati (*Nasua* sp.) from Morro do Ouro (ca. 4170–4640 cal BP), which were comparable (δ^13^C) and higher (δ^15^N) than most values for freshwater and marine-brackish fish from the region. Coatis feed on a range of animals (invertebrates and small vertebrates) and plants (e.g. leaves, fruits)^66, 67^, but such high δ^13^C and δ^15^N values would indicate a sustained intake of marine protein. Given the archaeological context, it is possible that some coatis benefited from anthropogenic food refuse during the occupation of the site, as they are known to do today^66^. It is also possible that coatis were kept and fed as pets or for food, as has been historically documented among some Indigenous groups in lowland South America^68, 69^. If so, this would represent the earliest evidence of the commensal interaction between coatis and humans in the Atlantic Forest, or pet-keeping in the region.

### Modelling dietary compositions

**Figure 5:**
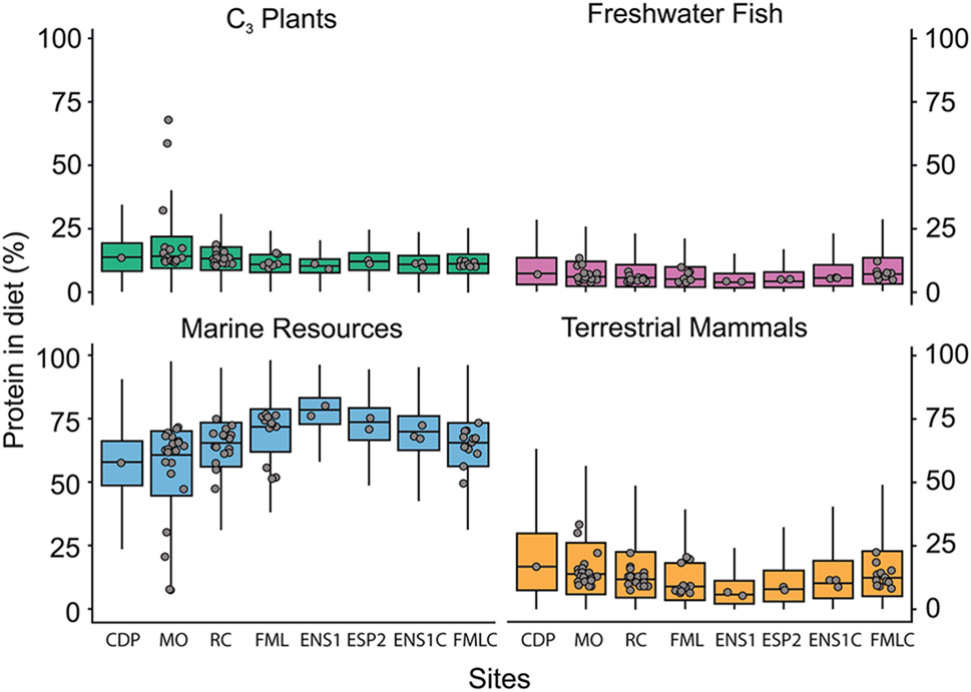
Box and whiskers plot summarizing the probability distribution (aggregated individual Markov chains) of the relative protein contributions from distinct food groups to individual diets in early and late non-ceramic contexts of Casa de Pedra (CDP), Morro do Ouro (MO), Rio Comprido (RC), Forte Marechal Luz (FML), Enseada I (ENS1) and Espinheiro II (ESP2), and ceramic contexts of Enseada I (ENS-C) and Forte Marechal Luz (FML-C). The horizontal lines correspond to the median and the hinges to the 25th (Q1) and 75th (Q3) percentiles. The whiskers extend from the hinge to the smallest or largest observation greater than or equal to − 1.5 * IQR or less than or equal to + 1.5 * IQR, respectively. Jitter plots represent the median values for each individual.

**Figure 6:**
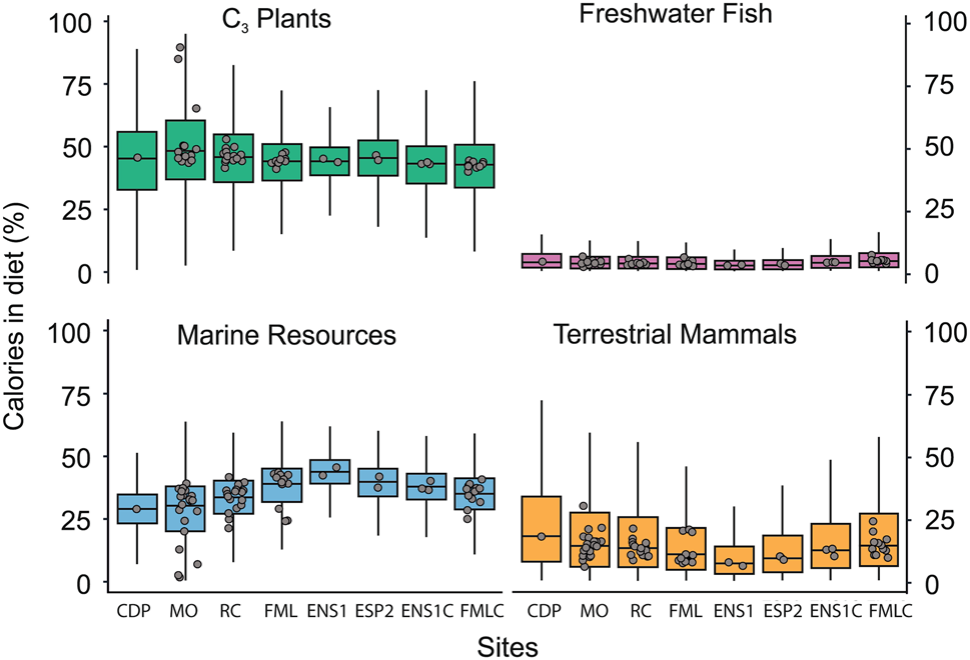
Box and whiskers plot summarizing the probability distribution (aggregated individual Markov chains) of the relative caloric contributions from distinct food groups to individual diets in early and late non-ceramic contexts of Casa de Pedra (CDP), Morro do Ouro (MO), Rio Comprido (RC), Forte Marechal Luz (FML), Enseada I (ENS1) and Espinheiro II (ESP2), and ceramic contexts of Enseada I (ENS-C) and Forte Marechal Luz (FML-C). The horizontal lines correspond to the median and the hinges to the 25th (Q1) and 75th (Q3) percentiles. The whiskers extend from the hinge to the smallest or largest observation greater than or equal to − 1.5 * IQR or less than or equal to + 1.5 * IQR, respectively. Jitter plots represent the median values for each individual.

Based on the newly generated faunal isotope baseline, the distribution of δ^13^C and δ^15^N values of individuals from non-ceramic (Enseada I, Espinheiro II, Forte Marechal Luz, Morro do Ouro, Rio Comprido, Casa de Pedra) and ceramic contexts (Enseada I, Forte Marechal Luz) in Babitonga Bay can be attributed to dietary regimes dominated by marine-brackish resources (Fig. 4). This is further corroborated by the outputs of BSIMMs, which estimated that marine-brackish resources were proportionally the main source of dietary proteins at all sites (Fig. 5), with relative median contributions ranging from 78% (Enseada I) to 58% (Casa da Pedra), followed by plants (10–14%), terrestrial mammals (6–17%) and ultimately by freshwater fish (4–7%). Although plant resources variably accounted for the majority of dietary calories (broadly equivalent to the whole diet) in all sites, with relative median contributions ranging from 48% (Morro do Ouro) to 43% (Enseada I and Forte Marechal Luz), marine-brackish resources also provided relatively high contributions to dietary calories, with median values ranging from 44% (Enseada I) to 29% (Casa de Pedra), followed by terrestrial mammals (7–18%) and to a lesser extent freshwater fish (2–4%) (Fig. 6). In general, uncertainties associated with individual estimates for both caloric and protein intake were less than 10%, indicating that model outputs were robust. As discussed for δ^13^C and δ^15^N values, an increasing trend in marine-brackish resource intake from early (Morro do Ouro) to late (Forte Marechal Luz) populations is visible in both dietary proteins and calories, as documented by previous studies in this region^40^. The differences were statistically significant for the model outputs (Supplementary Information 3), even though robust inter-site comparisons are hampered by the relatively small sample size of several collections (e.g. Casa de Pedra, Enseada I, Espinheiro II).

## Discussion

### Rise and fall of sambaquis in Babitonga bay

Our review of radiocarbon dates for coastal sites in the AFPB reaffirms that the bulk of sambaqui activities occurred predominantly between ca. 5500 and 2200 years ago, followed by a dramatic abandonment of several sites from ca. 2200 years ago. This pattern has been observed and discussed in detail for lagoonal systems in the south of Santa Catarina state^27, 46^ and here we show that it extends to Babitonga Bay and to other areas of the AFPB. We concur with others that this process may have been triggered by a combination of environmental and social drivers, involving substantial reorganization of coastal environments and key ecological niches, with an unprecedented cascade effect on the local carrying capacity and the long-standing social, political and ideological systems developed around large ceremonial sites.

Pollen, sedimentary, and geochemical data show significant changes in coastal and marine ecosystems in Babitonga Bay and adjacent coastal areas from ca. 2000 years ago due to a combination of coastal regression, increasing precipitation and intensification of cold fronts in the Late Holocene^56, 70–72^. The decrease in relative sea level from the Middle–Late Holocene (+ 2.6 m) to a lower elevation (+ 1 m) at ca. 1000 cal BP^45^ contributed to the progressive expansion of drylands and mangrove systems, potentially affecting spatial distribution and access to key resources (banks of intertidal and subtidal molluscs, fishing grounds, edible plants), as proposed for other regions^15, 21, 73^. The silting-up of the estuarine-lagoonal water bodies and the consequent loss of intercommunication between these systems would have certainly impacted the carrying capacity of ecological niches that had maintained large community-based sites for centuries prior. The downtrend of dated sites suggests a gradual decline of sambaquis from around 2500 cal BP, culminating in their sharp abandonment ca. 2200 cal BP. This event possibly represents a turning point in the community-based and resource-pooling nature of large ceremonial sites, which would have become unsustainable in most areas. As a result, several nucleated groups would have dissolved into smaller, dispersed and relatively independent social units organized in short-lived residential and logistic settlements that are notoriously less visible archaeologically. Although the radiocarbon dataset may be affected by a combination of taphonomic (e.g. site visibility and preservation) and research biases (e.g. more dates from non-ceramic sites), this population model would be supported by the increasing number of smaller and shallower sites along the coast of Santa Catarina state from ca. 2000 years ago^16–19, 25–29^.

As a result of this process, population density may have declined in several coastal areas, creating a new scenario of socio-cultural transformations and interactions with external groups^28, 46^. To date, genetic, osteological and archaeological data do not support the idea of ​​population replacement with the introduction of ceramic technology to the coast from ca. 1200 cal BP. Rather, the increasing presence of ceramic artefacts and evidence of population admixture reveal variable degrees of interaction between sambaquis and cultural groups from the highlands^28, 30, 44, 74–76^. The exact nature of these interactions is still a matter of debate, but it certainly involved the circulation of people, artefacts, ideas and worldviews.

### Fishing intensification in Babitonga bay

Evidence for economic intensification among prehistoric societies is a subject of contentious debate^77, 78^. Here it is alluded to by multiple lines of evidence showing increased fishing investment towards upper-trophic position species in Babitonga Bay. The initial socio-ecological context for this process was the unsustainability of the sambaquis’ nucleated systems of resource-pooling and sharing, and its fission into smaller, dispersed and potentially independent social units from ca. 2200 cal BP. Fishing intensification continued later by ceramic-using groups who were also producing distinctive fishing technologies^58, 79, 80^, in a new socio-ecological context of increased territoriality, as suggested by the first evidence of interpersonal violence^29, 81, 82^.

Our review of the stable carbon and nitrogen isotope composition of nearly 300 human individuals from non-ceramic and ceramic coastal sites reaffirms the central role of marine resources in sustaining populations along the southern Atlantic Forest coast of Brazil during the Late Holocene. The newly generated faunal baseline for pre-Columbian sites in Babitonga Bay, coupled with BSIMMs, indicate that the exploitation of marine-brackish resources provided the majority of dietary protein to coastal groups before and after the introduction of ceramic technology, being a fundamental subsistence strategy to enhance resilience during climate, environmental and social instability in the Late Holocene. Plants supplied most of the dietary calories, as documented by other lines of evidence^41, 43, 47^, and their importance to diet may have led to some local intensification^31^. However, in Babitonga Bay, individuals with mixed marine and C_3_ terrestrial diets or with diets dominated by terrestrial resources from C_3_ ecosystems were mostly found among early non-ceramic populations dated to the Middle-Late Holocene (e.g. Morro do Ouro, Rio Comprido, Casa de Pedra), while an increased reliance on high trophic level marine-brackish organisms appears to have occurred during the Late Holocene (Enseada I, Forte Marechal Luz, Espinheiros II). Crouch et al.^40^ analyzed the δ^13^C and δ^15^N values of several human individuals from the southern coast of Brazil and detected a significant increase in marine resource consumption with the spread of ceramics. Our results reveal this trend initiated prior to this introduction, in a late pre-ceramic phase contemporary with and prior to the decline of sambaquis.

The stable isotope data from Babitonga Bay suggest early non-ceramic populations secured marine proteins mainly from lower trophic level organisms, potentially including some degree of intertidal and subtidal molluscs. Although the dietary contribution of molluscs was not modelled due to complications in establishing stable isotope values for past specimens, their abundance in Middle-Late Holocene sites invites us to reconsider their role as source of protein, in addition to building material for site formation^25^. Studies have shown that marine molluscs may have offered a higher contribution to dietary proteins to prehistoric coastal societies than generally assumed^83, 84^. If so, a high intake of marine protein from intertidal and subtidal molluscs coupled with low trophic position fish could explain the relatively high δ^13^C and low δ^15^N values in early non-ceramic groups (e.g. Morro do Ouro, Rio Comprido, Casa de Pedra) compared to late non-ceramic (Enseada I, Forte Marechal Luz, Espinheiros II) and ceramic populations (Enseada I, Forte Marechal Luz). These later groups would have relied to a greater extent on fish, including high trophic level marine-brackish species.

The stable isotope results appear to be broadly supported by zooarchaeological studies indicating that Late Holocene groups, and particularly those using Taquara-Itararé ceramics, were fishing and hunting up the marine food web. For example, at the site of Enseada I in Babitonga Bay, fish become relatively more abundant in ceramic contexts (48%) compared to non-ceramic levels (32.4%), and involved the capture of high trophic level species (*Trichiurus lepturus*, *Lagocephalus laevigatus*, *Carcharias taurus*)^85^. At Forte Marechal Luz, deposits prior to and after the adoption of ceramics contained large amounts of sea mammal, bird, reptile, and fish remains, the latter including sharks and rays^58^. Risky and possibly costly to procure resources such as elasmobranchs (sharks, rays, and skates) were commonly targeted by some of these groups and, due to their individual biomass, may have made a much higher contribution to diet than previously considered^86, 87^. It remains unclear though if their exploitation also entailed aspects beyond their nutritional and artefactual^87^ value, such as prestige-related foraging goals^88^.

A review of the literature shows that the relative abundance of elasmobranchs over bony fish (%NISP), considering possible taphonomic biases, remained stable or increased in ceramic contexts compared to non-ceramic assemblages in the region. At Forte Marechal Luz, remains of elasmobranchs (e.g. *Aetobatus* sp., Selachii) accounted for 38% and 35% of fish remains in non-ceramic and ceramic contexts, respectively^58^. At Bupeva II, elasmobranchs included Rajiformes, *Carcharhinus isodon*, *Galeocerdo cuvier*, *Carcharodon carcharias*, *Isurus oxyrinchus*, *Carcharias taurus*, and *Sphyrna* sp., represented 27% of the fish remains in non-ceramic contexts, while in ceramic deposits it rose to 42%^85^. In Enseada I, elasmobranchs included Rajiformes, *Galeocerdo cuvier*, *Prionace glauca*, *Carcharodon carcharias*, *Carcharias taurus*, and *Sphyrna* sp., which contributed to 1.6% and 3% in non-ceramic and ceramic contexts, respectively^85^. In other non-ceramic sites elasmobranch remains were relatively smaller components of fish assemblages, from 4% (Cubatão I) to 1% (Espinheiros II)^22, 89, 90^. Moving south, remains of top predators such as fur seals (*Arctocephalus australis*), dolphins (*Delphinus delphis*, *Tursiops truncatus*) and sharks (*Carcharias taurus*, *Carcharodon carcharias*, *Galeocerdo cuvier*), among others, were found in ceramic deposits of Praia das Laranjeiras II (Camboriú)^79^. At the ceramic site of Rio do Meio (Florianópolis), a large variety of fish, including a remarkable diversity of sharks (*Carcharhinus plumbeus*, *C. obscurus*, *C. leucas*, *C. brachyurus*, *Rhizoprionodon* sp., *Carcharodon carcharias*, etc.) were exploited^86, 87, 91^. A similar faunal record was documented for the ceramic site of Tapera, also in Florianópolis, which included sharks (Odontaspis, *Prionace glauca*, *Galeocerdo cuvier*) and sea mammals (Otariidae, *Tursiops truncatus*)^92^. Further south, ceramic-using groups at Galheta IV (Laguna) targeted a range of fish, sea mammals (Otariidae), sharks (e.g. *Carcharias taurus*, *Carcharodon carcharias*, *Sphyrna* sp., *Prionace* sp.), rays (Dasyatidae, *Rhinoptera* sp.) and sea birds (*Thalassarche* sp.)^93^.

Human osteological records provide evidence for increasing interpersonal violence among ceramic-using populations^29, 81, 82^, possibly in a context of competition for resources and increased territoriality^94, 95^. This may have prompted technological innovation or adoption, represented by the appearance of more sophisticated fishing technologies such as baited hooks manufactured from mammal bones in Babitonga Bay and adjacent coastal areas from ca. 1200 years ago^58, 79, 80^. Mostly found in contexts with ceramic artefacts (i.e. in Itacoara, Enseada I, Forte Marechal Luz, but also Laranjeiras II, Rio do Meio and potentially Cabeçudas in the north of Santa Catarina state^19, 58, 79^), these hooks reflect attempts to increase fishing efficiency and expand fishing to new ecological niches and fishing grounds. Baited hooks are not contingent upon the visibility of the target, and thus are an ideal implement for expanding the fisheries to deeper waters, including offshore habitats, and to pelagic species. Multiple hooks can be simultaneously operated by a single individual, potentially increasing individual fishing efficiency. Conceivably, other fishing hooks partially composed of bone and perishable materials (wood) may have been used in earlier times, as suggested by numerous bone points in sambaqui sites^11^, but their functional interpretation remains a matter of debate. The importance of aquatic resources is also reflected in the use of ceramic artefacts, as revealed by residues of aquatic organisms in Taquara-Itararé pots from coastal sites^30, 96–98^.

Overall, these lines of evidence support the view that coastal adapted populations of the Atlantic Forest are examples of how fishing intensification was a possible outcome of environmental and social instability during the Late Holocene. While we recognize that demographic trends and dietary estimates can be further refined with increasing radiocarbon dates and stable isotope analysis of faunal and plant baselines, the results presented herein reinforce the central role of coastal resources to significant cultural and demographic processes in pre-Columbian South America.

## Materials and methods

### Modelling the chronology of coastal non-ceramic and ceramic sites

The radiocarbon dates used in this study were compiled from the Brazilian Radiocarbon Database^99^. The dates were generated from a range of archaeological materials (marine shell, human and faunal bone, charcoal) and only those dates which reported the material dated were selected for use. We collated 399 radiocarbon dates from non-ceramic sambaqui sites (n = 187) along the coasts of São Paulo, Paraná, Santa Catarina and Rio Grande do Sul (southeastern and southern Brazil), a macroregion covered by the southern Atlantic Forest and the Pampa Biome (AFPB). This region has the highest concentration of shell mounds of the Brazilian coast, and previous studies have shown some long-standing cultural affinities between groups occupying these regions during the Late Holocene^11, 100, 101^. We also collected 27 radiocarbon dates associated with ceramic artefacts attributed to the Taquara-Itararé tradition from sites (n = 13) along the coast of Santa Catarina state, the only region with available ^14^C dates for coastal sites with Taquara-Itararé pots. Overall, the dates represent heterogeneous events in space and time (Supplementary Information 1). The density distributions of the radiocarbon dates were modelled for non-ceramic and ceramic contexts using a Kernel Density Estimation (KDE) model^50^ in OxCal v. 4.4^102^, with the kernel and factor defaults set to N(0,1) and U(0,1), respectively, and outputs rounded to 50 years. This approach integrates the variation in the number of dated events and the chronological uncertainty through-time, producing smooth estimates (removing high frequency noise) with associated uncertainties^50, 103^. The quality and reliability of model outputs were assessed using agreement indexes (A_model_) with an acceptable threshold of > 60. Terrestrial samples (charcoal, seeds, wood) were calibrated using the 100% atmospheric calibration curve for the southern hemisphere, SHCal20^104^. Marine samples (shells, otoliths) were calibrated using the 100% Marine20 curve^105^, applying an estimated average local marine radiocarbon reservoir correction value (ΔR) of − 126 ± 29 for the coasts of São Paulo, Paraná, Santa Catarina and Rio Grande do Sul, generated from eight reference points between latitudes 32.00°S and 23.73°S^106–108^ according to the Marine Reservoir Correction database (http://calib.org/marine/). Given the usually high contribution of marine carbon to bone collagen of individuals in this region, the radiocarbon dates on human bone collagen were modelled using a mixed curve (SHCal20 and Local Marine curve) using the same ΔR value reported above. We considered the average relative contribution of marine carbon to collagen derived from BSIMMs (see below). Specifically, for sites from Babitonga Bay analysed here for δ^13^C and δ^15^N values (Enseada I, Morro do Ouro, Rio Comprido, Forte Marechal Luz), we computed the average relative contribution of marine carbon to collagen obtained from several individuals from the sites. For individuals with no prior information on the relative contribution of marine carbon to collagen, we assumed a fixed value of 52 ± 9%, which is the average estimated contribution obtained from 73 individuals from sites in Babitonga Bay.

### Review of human and faunal stable isotope data

We reviewed the literature and collated the bulk collagen δ^13^C and δ^15^N values of human and faunal samples associated with coastal non-ceramic and ceramic contexts in the southeast and south of Brazil. Published δ^13^C and δ^15^N values were selected based on the following criteria: (1) the original publication presented a detailed collagen extraction protocol, or (2) the original publication presented a summarized protocol and references for detailed extraction methods, and (3) the original publication presented collagen samples with atomic C:N molar ratios between 2.9 and 3.6 (with few exceptions), which is a conventional quality control for assessing contamination (e.g. humic acids) and deterioration of bone collagen^109–111^. Other quality criteria include minimum wt% of extracted collagen (1%), wt%C (13%) and wt%N (4.8%)^110, 111^, but these were seldom reported in the archaeological literature for the region. When collagen of the same individual had been analysed by more than one study, the isotope values and collagen atomic composition were averaged (Supplementary Information 2).

### Faunal selection from Babitonga bay for stable isotope analysis

In order to expand the archaeological faunal baseline to Babitonga bay we sampled a range of faunal remains (n = 143) from non-ceramic and ceramic contexts dated between ca. 6000 and 1350 cal BP^22^ (Supplementary Information 2). The permits for stable isotope analyses were obtained from the Instituto do Patrimônio Histórico e Artístico Nacional (IPHAN, protocol no. 0510.000990/2018-26, 01510.000676/2021-49). The samples consisted of marine-brackish water fish (n = 80), terrestrial mammals (n = 22), freshwater fish (n = 20) and sea mammals (n = 21). Whenever possible, specimens were selected to represent individual animals by sampling the same anatomical element. Fish and terrestrial mammal remains were identified using modern reference collections of the Laboratório de Arqueologia e Patrimônio Arqueológico (Universidade da Região de Joinville, Brazil) and Museu Arqueológico de Sambaqui de Joinville (Brazil). Several samples could be identified to species level, but in most cases only genus and family could be determined. Terrestrial mammals included peccary (*Tayassu* sp.), capybara (*Hydrochoerus* sp.) and coati (*Nasua* sp.). Marine-brackish water fish included whitemouth croaker (*Micropogonias furnieri*), barred grunt (*Conodon nobilis*), cutlassfish (*Trichiurus* sp.), archosargus (*Archosargus* sp.), snook (*Centropomus* sp.), puffer fish (*Lagocephalus* sp. or *Sphoeroides* sp.) and catfish (Ariidae). Freshwater fish were represented by catfish (Loricariidae) and hoplias (*Hoplias* sp.). These are the most common and abundant species in pre-Columbian coastal sites in the region^22, 49^, and are expected to have made a substantial contribution to pre-Columbian diets. A larger sample size would be recommended to account for isotopic variability that may arise from local to regional scale processes, such as short-term climate variability^112, 113^ and overfishing^114^. Such factors are potential sources of uncertainty in our faunal baseline and model outputs. However, taxonomically and chronologically well-resolved faunal remains are of limited availability in this region.

Marine mammal samples were identified through collagen peptide mass fingerprinting following a modified protocol to that described in^115^. Briefly, extracted collagen from stable isotope analysis was subsampled (0.3–0.7 mg) and resuspended in ammonium bicarbonate buffer (NH_4_HCO_3_, pH 8) then digested with 0.4 µg of trypsin at 37 °C overnight. Samples were acidified to 0.1% trifluoroacetic acid (TFA) to stop the trypsin, then purified using C18 resin ZipTip pipette tips (EMD Millipore). 1 µl of sample was combined with 1 µl of matrix solution (α-cyano-hydroxycinnamic acid) and run in triplicate along with calibration standards on a Bruker ultraflex III MALDI TOF/TOF mass spectrometer. Spectra were analysed using mMass software^116^ and species were determined based on the list of m/z markers presented in^115, 117, 118^. Identified taxa included humpback whale (*Megaptera novaeangliae*), right whale (most likely *Eubalaena australis* based on geographic context) and oceanic dolphins (Delphinidae) (Supplementary Information 2).

### Collagen extraction and stable isotope analysis

The bone collagen of 143 faunal remains from Babitonga Bay were extracted at the BioArCh facilities of the Department of Archaeology, University of York (UK). Collagen was also extracted from one human sample (rib) from the non-ceramic site of Casa de Pedra (Babitonga Bay). Bones were cleaned mechanically to remove surface contaminants, with minimal cleaning carried out on fish samples due to their small size. The protocol for collagen extraction followed a modified Longin method^119^, details for which can be found in previous studies^120^. In short, shards of bone, and in some cases entire fish bones, (ca. 60–500 mg) were demineralized using 0.6 M HCl at 4 °C for several days, then rinsed with ultrapure H_2_O (Milli-Q) and gelatinized with 0.001 M HCl (pH 3) at 80 °C for 48 h. Samples were then ultrafiltered (30 kDa, Amicon® Ultra-4 centrifugal filter units; Millipore, MA, USA), frozen and freeze dried. Duplicate collagen samples (0.5 mg) were combusted to obtain CO_2_ and N_2_ with a Sercon GSL system attached to a Sercon 20–22 mass spectrometer (Sercon, Crewe, UK). Standardization was carried out using in-house fish gelatine: δ^13^C − 15.5 ± 0.1‰, δ^15^N 14.3 ± 0.2‰ that served as a check standard throughout the analysis; while cane sugar IA-R006: δ^13^C − 11.8 ± 0.1‰, caffeine IAEA 600: δ^13^C − 27.8 ± 0.1‰, δ^15^N 0.8 ± 0.1‰, and ammonium sulfate IAEA N2: δ^15^N 20.4 ± 0.2‰) were used as calibration standards following a two-point calibration procedure. Precision was < 0.2‰ (1σ) for both δ^13^C and δ^15^N values. δ^13^C and δ^15^N values in this paper are reported as a normalized mean of duplicate results, only for those samples which fell within the acceptable ranges for collagen quality control (90 out of 143). Uncertainty was calculated with the Kragten spreadsheet^121, 122^. In our newly generated dataset, the C:N molar ratios were considered acceptable when falling between 2.9 and 3.6, with few exceptions^109^. However it is important to mention that following recently published quality criteria, three of our samples (MO 037; MO 034; CP5) could have their original δ^13^C values altered by up to 1‰^123^.

### Statistical analysis and Bayesian stable isotope mixing models

Dietary regimes based on multiple food sources can be affected by large uncertainties due to differences in food macronutrient composition (protein, carbohydrate, lipids) and their respective isotopic values, and also due to the varying degree of routing of dietary compounds (e.g. amino acids) to consumer tissue and the diet-to-tissue isotopic offsets. Recent developments in Bayesian Stable Isotope Mixing Models (BSIMMs) allow for dietary estimations that express food source contribution probability distributions, while taking into account the uncertainties reported above^124, 125^. Therefore BSIMMs was employed to estimate the relative contribution of different food sources to individual diets in Babitonga Bay.

We selected previously measured bulk collagen δ^13^C and δ^15^N values of 73 human individuals associated with non-ceramic (n = 56) and ceramic (n = 15) contexts. All individuals were adults except two individuals from Rio Comprido (RC2A, RC42B) whose age was estimated at 6 and 8 years, respectively^31, 34^, and were not expected to retain any breastfeeding signal. Non-ceramic contexts included the sites of Espinheiros II, Enseada I, Morro do Ouro, Rio Comprido, Forte Marechal Luz and Casa de Pedra; ceramic contexts included the sites of Enseada I and Forte Marechal Luz. Sample information and details on archaeological contexts can be found in^22, 31, 33, 40^. We used BSIMMs in FRUITS 3.1, opting for a concentration-dependent and routed model where it assumes that nitrogen isotopes are 100% sourced from proteins, while carbon isotopes can to some degree derive from carbohydrates and lipids, depending on diet^126^. FRUITS has previously been used with pre-Columbian coastal populations in lowland eastern South America^14, 31, 127^, thus offering a common framework for regional comparison of model outputs. An advantage of FRUITS is that it allows for modelling dietary routing (e.g. proteins, energy) while considering uncertainties at different stages of the modelling. For example, here we considered that dietary proteins contributed to 74 ± 4% of bulk collagen carbon, while lipids and carbohydrates (here aggregated as energy) provided the remaining 26%^128^. The model assumptions and parameters were essentially the same as used by Pezo-Lanfranco et al.^31^, with the difference that isotopic composition of macronutrients in the faunal baseline were calculated from the average bulk collagen δ^13^C and δ^15^N values of the terrestrial mammals, freshwater fish and marine-brackish resources (fish, sea mammals) analysed in this study. For terrestrial mammals: − 2‰ (∆^13^C_protein-collagen_), − 8‰ (∆^13^C_lipids-collagen_) and + 2‰ (∆^15^N_protein-collagen_); freshwater fish: − 1‰ (∆^13^C_protein-collagen_), − 7‰ (∆^13^C_lipids-collagen_) and + 2‰ (∆^15^N_protein-collagen_); marine-brackish fish and sea mammals: − 1‰ (∆^13^C_protein-collagen_), − 7‰ (∆^13^C_lipids-collagen_) and + 2‰ (∆^15^N_protein-collagen_). Intertidal and subtidal molluscs (notably *Anomalocardia flexuosa*) were not modelled here due to complications in establishing isotope values for their macronutrients in past specimens.

For plants we used the average δ^13^C (− 29.2 ± 3.0‰) and δ^15^N (+ 1.1 ± 2.0‰) values of modern fruits (n = 30), roots (n = 5) and palm-heart (n = 13) collected in national parks in the southeastern Atlantic Forest between 2010 and 2012^129^. Plant δ^13^C values were corrected for the Suess effect (+ 2‰) using the δ^13^C value of the atmosphere in 2010 (− 8.4‰)^130^. Plant macronutrients δ^13^C values were then corrected for the offsets of − 2‰ (∆^13^C_bulk-protein_) and + 0.5‰ (∆^13^C_bulk-carbohydrate_). We assumed that the δ^15^N value of plant protein was the same as the average bulk plant δ^15^N value. A conservative uncertainty of 1‰ was used for the δ^13^C and δ^15^N values of macronutrients. Diet-to-collagen δ^13^C offset (+ 4.8 ± 0.5‰) and δ^15^N offset (+ 5.5 ± 0.5‰) were taken from Fernandes et al.^131^. Dietary estimations considered a conservative acceptable range for protein intake of > 5% and < 45%^132^.

Box and whiskers plots summarizing the posteriors of FRUITS estimates (Markov chains) were generated using the R statistical software environment (version 4.0.3) and the package ggplot2 (version 3.3.2)^133^, with McGill et al.^134^ variations of Box Plots. The horizontal lines correspond to the median and the hinges to the 25th (Q1) and 75th (Q3) percentiles. The whiskers extend from the hinge to the smallest or largest observation greater than or equal to − 1.5 * IQR (interquartile range) or less than or equal to + 1.5 * IQR, respectively; outliers were excluded.

Due to the large volume of data (730k) generated for each individual in Markov chains and by the central limit theorem^135^, estimates in the Markov chain tend toward a normal distribution, even though the original isotopic variables themselves were not normally distributed. For this reason, comparison of model outputs (Markov chains) from each context were performed using non-parametric tests, using Kruskal–Wallis with Dunn’s multiple comparison post hoc test in dplyr (version 1.0.1). Comparisons of δ^13^C and δ^15^N values between humans and faunal samples were performed using parametric (one-way ANOVA) or non-parametric (Kruskal–Wallis) tests (α = 0.05), after checking for normal distribution with Shapiro–Wilk test for normality (α = 0.05) (Supplementary Information 3). Tests were performed in IBM SPSS Statistics 25.

## Supplementary Information

Supplementary Information 1.

Supplementary Information 2.

Supplementary Information 3.

Supplementary Information 4.

Supplementary Information 5.
