# Supplementary material for: Fishing intensification as response to Late Holocene socio-ecological instability in southeastern South America
Source: Sci Rep. 2021 Dec 6;11:23506. doi: 10.1038/s41598-021-02888-7 (PMC8648744; doi:10.1038/s41598-021-02888-7)
Supplement: Supplementary file 5 — Supplementary Information 5. [file 41598_2021_2888_MOESM5_ESM.docx]

**Fish-based economies fuelled population growth in southeastern South America during the Late Holocene**

Alice Toso^1,2^, Ellen Hallingstad^2^, Krista McGrath^1,2^, Thiago Fossile^1^, Christine Conlan^2,3^, Jessica Ferreira^4^, Dione da Rocha Bandeira^5^, Paulo César Fonseca Giannini^6^, Simon-Pierre Gilson^7^, Lucas de Melo Reis Bueno^8^. Murilo Quintans Ribeiro Bastos^9^, Fernanda Mara Borba^5^, Adriana M. P. do Santos^5^, André Carlo Colonese^1,2*^

1 Department of Prehistory & Institute of Environmental Science and Technology (ICTA), Universitat Autònoma de Barcelona, Bellaterra, 08193, Spain

2 BioArCh, Department of Archaeology, University of York, York, YO10 5DD, UK

3 Department of Archaeology, Simon Fraser University, Education Building 9635, 8888 University Dr., Burnaby, BC V5A 1S6, Canada

4 Departamento de Ciências Biológicas - Meio Ambiente e Biodiversidade, Universidade da Região de Joinville, Rua Paulo Malschitzki 10, Zona Industrial Norte, 89219-710, Joinville, Santa Catarina, Brazil

5 Museu Arqueológico de Sambaqui de Joinville; Programa em Patrimônio Cultural e Sociedade, Universidade da Região de Joinville, Joinville, Brazil

6 Instituto de Geociências, Rua do Lago, 562, Universidade de São Paulo, São Paulo, Brazil

7 Instituto de Ciências Humanas e da Informação, Universidade Federal do Rio Grande

8 Departamento de História, Laboratório de Estudos Interdisciplinares em Arqueologia (LEIA), Universidade Federal de Santa Catarina.

9 Departamento de Antropologia, Museu Nacional, Universidade Federal do Rio de Janeiro, Rio de Janeiro, Brazil

*Corresponding author


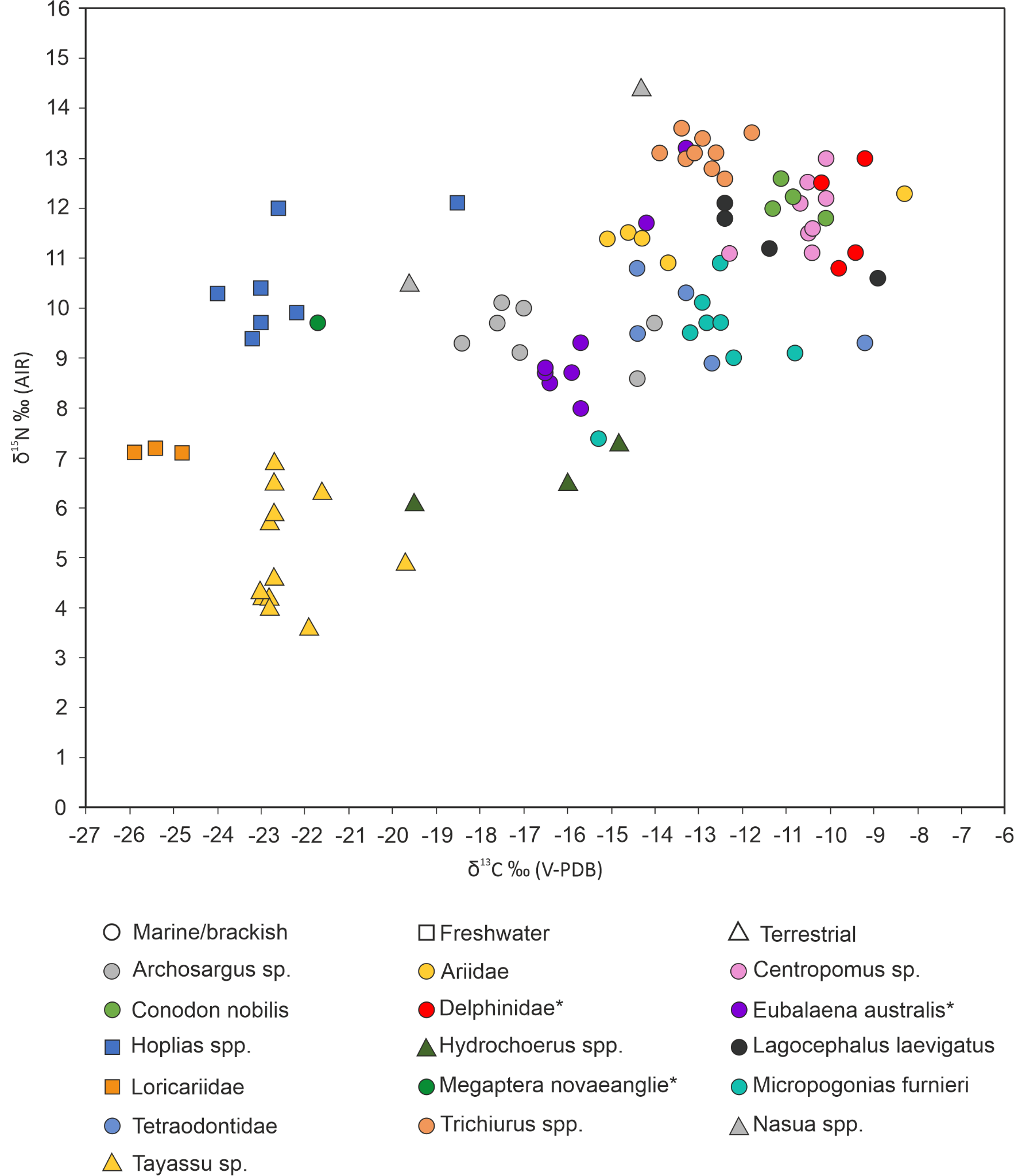


**δ**^13^C and δ^15^N values of faunal remains from Babitonga Bay. Samples with * have been identified using ZooMS. See SI 2 for details.

Marine-brackish water fish (n = 50; Morro do Ouro, Cubatão I, Bupeva II, Casa de Pedra) had δ^13^C values ranging from -18.4 to -8.3 ‰ (average -12.8 ± 2.3 ‰), and δ^15^N values from +7.4 to +13.6 ‰ (average +11.1 ± 1.5 ‰). Marine-brackish water fish had δ^13^C values reflecting assimilated carbon from benthic microalgae and marine phytoplankton [[1]](https://paperpile.com/c/fSY4O9/SkdIf). The δ^15^N values indicate distinct trophic positions, from piscivore-invertivore taxa (e.g. Trichiurus sp.) to omnivore species feeding invertebrates and occasionally fish (e.g. Tetraodontidae, Micropogonias furnieri), depending on age and season, but in general they fall into the range of modern fish from Babitonga Bay [[2]](https://paperpile.com/c/fSY4O9/Hb1v1). The relatively low δ^15^N values are consistent with the trophic ecology of fish communities from estuarine-mangrove systems which are dominated by small-bodied and juvenile individuals [[3,4]](https://paperpile.com/c/fSY4O9/luI7l+9pl3h). Juvenile specimens inhabiting mangrove systems are expected to have lower δ^15^N values than their adult counterparts from deeper habitats (ocean, coastal bays) due to ontogenetic differences in feeding behaviour and trophic positions [[5,6]](https://paperpile.com/c/fSY4O9/71Ixx+X2NTm). Freshwater fish (n = 10, Itacoara) had δ^13^C values ranging -25.9 to -18.5 ‰ (average -23.3 ± 2.0 ‰), and δ^15^N values ranging from +7.1 to +12.1 ‰ (average +9.5 ± 1.8 ‰). The δ^13^C values are consistent with the influence of organic matter derived from C_3_ vegetation and phytoplankton in freshwater systems [[1]](https://paperpile.com/c/fSY4O9/SkdIf). Terrestrial faunal remains (n = 17; Morro do Ouro, Cubatão I, Itacoara) produced δ^13^C values ranging from -23.0 to -14.3 ‰ (average -20.7 ± 2.9 ‰) and δ^15^N values ranging from +3.6 to +14.4 ‰ (average +6.2 ± 2.6 ‰).

Marine mammal samples (n = 13; Morro do Ouro, Bupeva II and Casa de Pedra) identified through collagen peptide mass fingerprinting included Eubalaena australis, Megaptera novaeangliae and oceanic dolphins (Delphinidae). Studies suggest that sea mammals may have been only occasionally consumed by groups in this region [[7]](https://paperpile.com/c/fSY4O9/YqYNu). Their δ^13^C and δ^15^N values comprehensively ranged from -21.7 to -9.2 ‰ (average -14.2 ± 3.7 ‰) and +8 to +13.2 ‰ (average +10.3 ± 1.83 ‰) respectively. The δ^13^C and δ^15^N values of right whale (Eubalaena australis) from Morro do Ouro (n = 4) and Bupeva II (n = 4) fall into the range of their modern subpopulation in southern Brazil [[8]](https://paperpile.com/c/fSY4O9/bQ9j7), after considering the δ^13^C and δ^15^N offsets between muscle and bone collagen [[9]](https://paperpile.com/c/fSY4O9/xGEC), and the Suess effect [[10]](https://paperpile.com/c/fSY4O9/1TkgT) on modern δ^13^C values (ca. -1.5 to -2 ‰). Similarly, the δ^13^C and δ^15^N values of humpback whale (Megaptera novaeangliae) from Casa de Pedra are compatible with values observed in modern specimens feeding predominantly on arctic krill [[11]](https://paperpile.com/c/fSY4O9/spAw0). By contrast, the δ^13^C and δ^15^N values of oceanic dolphins (Delphininae) from Morro do Ouro are higher and lower respectively to values from modern dolphins (Sotalia guianensis, Pontoporia blainvillei) from Babitonga Bay and adjacent coastal areas [[2]](https://paperpile.com/c/fSY4O9/Hb1v1). This suggests that dolphins captured at Morro do Ouro ca. 4300 years ago fed on marine organisms at a lower trophic position compared to modern local populations.

**Reference**

1. [Garcia AM, Hoeinghaus DJ, Vieira JP, Winemiller KO. Isotopic variation of fishes in freshwater and estuarine zones of a large subtropical coastal lagoon. Estuar Coast Shelf Sci. 2007;73: 399–408.](http://paperpile.com/b/fSY4O9/SkdIf)

2. [Hardt FAS, Cremer MJ, Tonello Junior AJ, Bellante A, Buffa G, Buscaino G, et al. Use of carbon and nitrogen stable isotopes to study the feeding ecology of small coastal cetacean populations in southern Brazil. Biota Neotrop. 2013;13: 90–98.](http://paperpile.com/b/fSY4O9/Hb1v1)

3. [Gerhardinger LC, Herbst DF, da Cunha SMB, de Paula Costa MD. Diagnóstico da Ictiofauna do Ecossistema Babitonga. Revista CEPSUL. 2020;9: eb2020001–eb2020001.](http://paperpile.com/b/fSY4O9/luI7l)

4. [Laegdsgaard P, Johnson C. Why do juvenile fish utilise mangrove habitats? J Exp Mar Bio Ecol. 2001;257: 229–253.](http://paperpile.com/b/fSY4O9/9pl3h)

5. [Stuthmann LE, Castellanos-Galindo GA. Trophic position and isotopic niche of mangrove fish assemblages at both sides of the Isthmus of Panama. Bull Mar Sci. 2020;96: 449–468.](http://paperpile.com/b/fSY4O9/71Ixx)

6. [Romanuk TN, Hayward A, Hutchings JA. Trophic level scales positively with body size in fishes: Trophic level and body size in fishes. Glob Ecol Biogeogr. 2011;20: 231–240.](http://paperpile.com/b/fSY4O9/X2NTm)

7. [de Castilho PV. Utilization of cetaceans in shell mounds from the southern coast of Brazil. Quat Int. 2008;180: 107–114.](http://paperpile.com/b/fSY4O9/YqYNu)

8. [Vighi M, Borrell A, Crespo EA, Oliveira LR, Simões-Lopes PC, Flores PAC, et al. Stable isotopes indicate population structuring in the southwest Atlantic population of right whales (Eubalaena australis). PLoS One. 2014;9: e90489.](http://paperpile.com/b/fSY4O9/bQ9j7)

9. [Sholto-Douglas AD, Field JG, James AG, van der Merwe NJ. 13C/12C and 15N/14N isotope ratios in the Southern Benguela Ecosystem: indicators of food web relationships among different size–classes of plankton and pelagic fish; differences between fish muscle and bone collagen tissues. Mar Ecol Prog Ser. 1991;78: 23–31.](http://paperpile.com/b/fSY4O9/xGEC)

10. [Hellevang H, Aagaard P. Constraints on natural global atmospheric CO2 fluxes from 1860 to 2010 using a simplified explicit forward model. Sci Rep. 2015;5: 17352.](http://paperpile.com/b/fSY4O9/1TkgT)

11. [Eisenmann P, Fry B, Holyoake C, Coughran D, Nicol S, Bengtson Nash S. Isotopic Evidence of a Wide Spectrum of Feeding Strategies in Southern Hemisphere Humpback Whale Baleen Records. PLoS One. 2016;11: e0156698.](http://paperpile.com/b/fSY4O9/spAw0)

**Supplementary tables legends**

Supplementary Information 1 (SI_1_Radiocarbon dates and model outputs). Radiocarbon dates and KDE model outputs.

Supplementary Information 2 (SI_2).  δ^13^C and δ^15^N values of human and faunal remains included in study.

Supplementary Information 3 (SI_3_Chain statistics). Probability distribution (aggregated individual Markov chains) of the relative protein and calories contributions with statistical results.

Supplementary Information 4 (SI_4_ Fruits report). Bayesian stable isotope mixing model output.

Supplementary Information 5 (SI_5 Faunal remains from Babitonga Bay). δ^13^C and δ^15^N values of faunal remains from Babitonga Bay analysed for this study.
